# Supplementary material for: Recovery of Spinal Walking in Paraplegic Dogs Using Physiotherapy and Supportive Devices to Maintain the Standing Position
Source: Animals (Basel). 2023 Apr 19;13(8):1398. doi: 10.3390/ani13081398 (PMC10135265; doi:10.3390/ani13081398)
Supplement: Supplementary file 1 [file animals-13-01398-s001.zip › Figure S1.pdf]

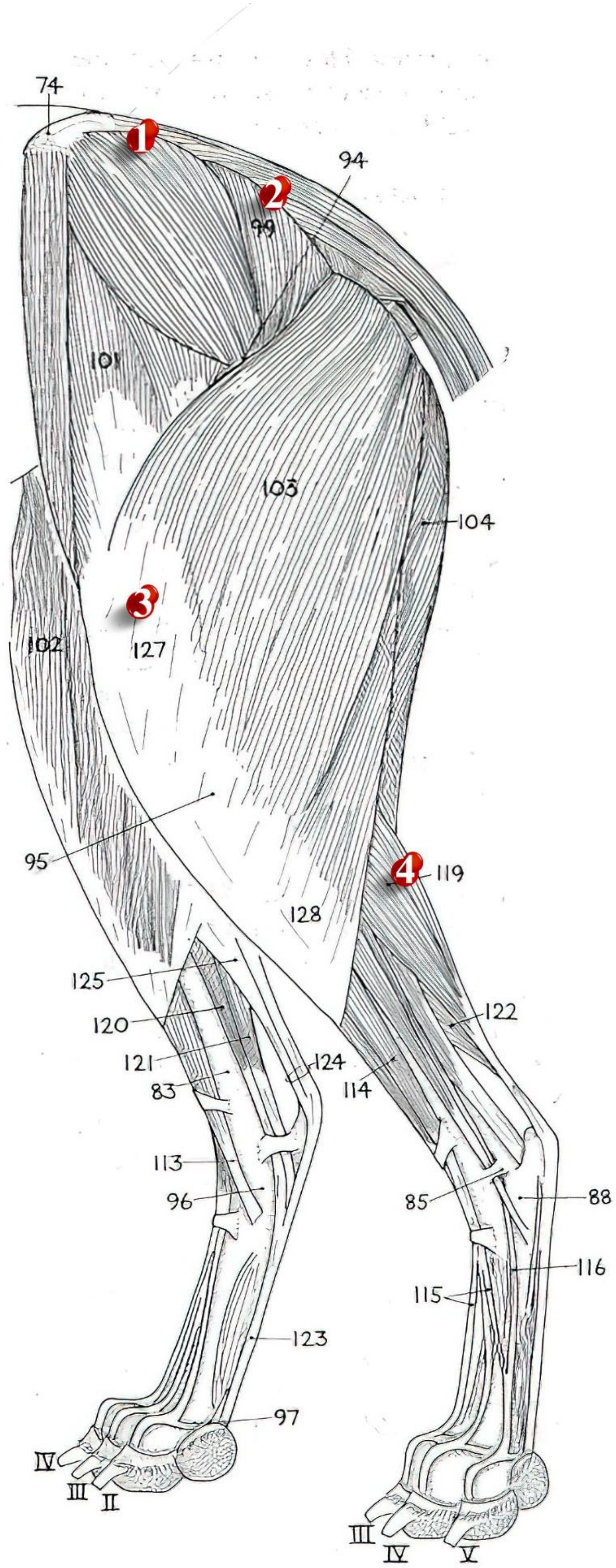

Figure S1. Motor points in dog (after Goody, 59)

1, Middle gluteal muscle – origin ilial wing, sacrum, and 1st lumbar vertebre – insert greater trochanters

2 Superficial gluteal muscle – Origin Gluteal fascia and sacrum – insert greater and third trochanters

3. Cranial tibial muscle – origin lateral condyle of tibia – insert tarsal and metatarsal bones

4 Gastrocnemius muscle – origin distal on femur – insert calcaneal tuber (achilles tendon)

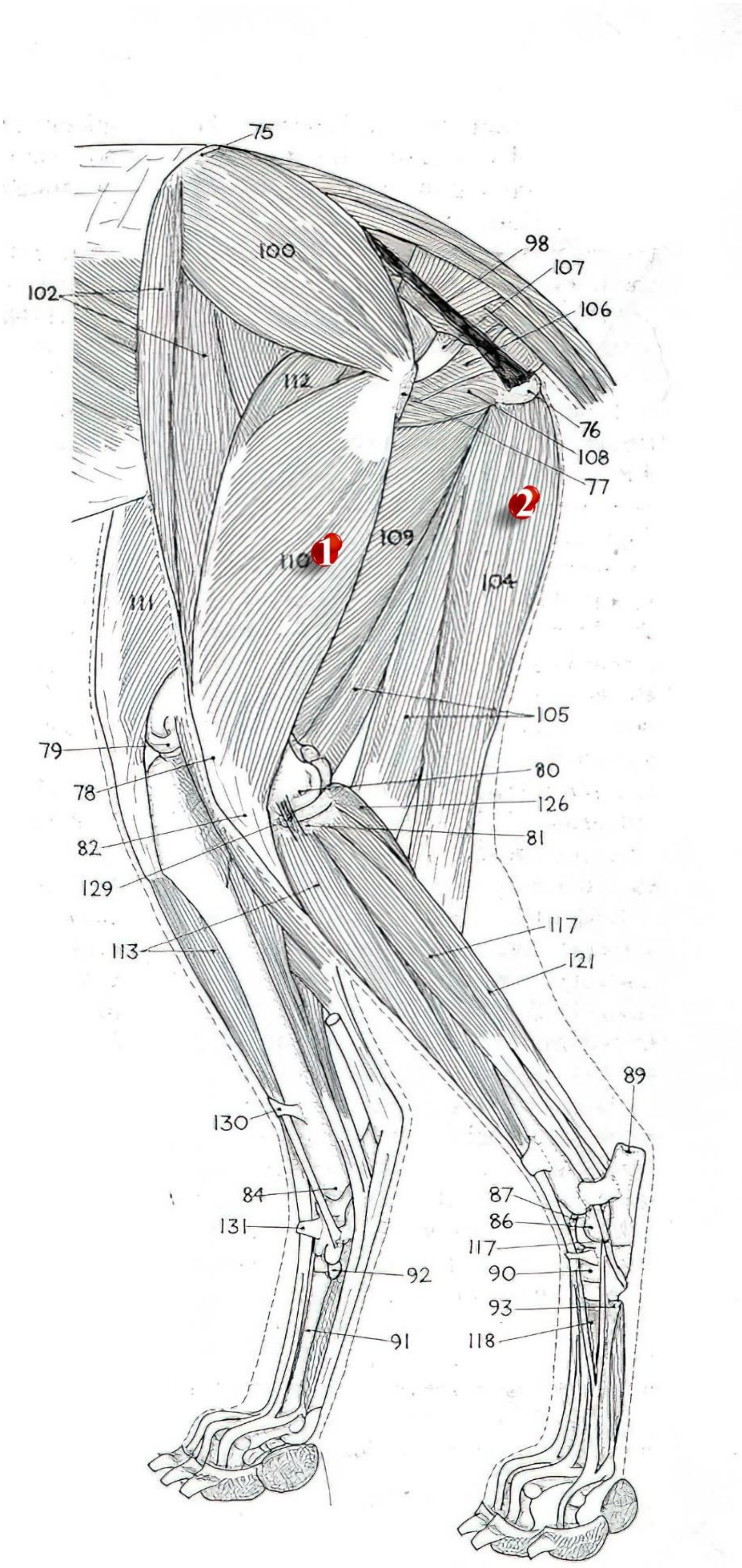

1. Lateral vastus quadriceps – Origin Cranial surface of femur – insert patella, tibial tuberosity

2. Semitendinous muscle – origin vertebral head – insert medial condyle of femur and tibia
