# Supplementary material for: Recovery of Spinal Walking in Paraplegic Dogs Using Physiotherapy and Supportive Devices to Maintain the Standing Position
Source: Animals (Basel). 2023 Apr 19;13(8):1398. doi: 10.3390/ani13081398 (PMC10135265; doi:10.3390/ani13081398)
Supplement: Supplementary file 1 [file animals-13-01398-s001.zip › Table S1.pdf]

Table S1. Cohort of dogs population involved in the study

Nr Crt	Breed	Weight	Age	Lesion Localization	Lesion
**Dogs that developed spinal walking**					
1	Bichon	5.2	4.25	T13 - L1	IVHD
2	Shih Tzu	9.1	10.5	L1 - L2	IVHD
3	Bichon	2	0.25	T13 - L1	Trauma
4	Yorkshire Terrier	3.5	5.5	T13 - L1	Trauma
5	Pekingese	7.3	6.25	T13 - L1	IVHD
6	Crossbreed	9.6	2	T11 - T12	Trauma
7	Pekingese	6.5	5.5	T11 - T12	IVHD
8	Crossbreed	4	6.75	T13 - L1	IVHD
9	Crossbreed	5.4	5	T13 - L1	IVHD
10	Teckel	9.2	6.25	T9 - T10	IVHD
11	Crossbreed	7.4	3.5	T9 - T10	IVHD
12	French Bulldog	13.1	2.75	T10 - T11	IVHD
13	Bulldog	15.7	8.25	T10 - T11	IVHD
14	Crossbreed	11.3	1	T13 - L1	IVHD
15	Peke-a-Pap	3.3	2	T13 - L1	IVHD
16	Shih Tzu	4.1	3.5	T13 - L1	IVHD
17	Bichon	6.5	6	T13 - L1	IVHD
18	Crossbreed	9.6	3	T13 - L1	IVHD
19	Poodle	11.2	0.75	T13 - L1	IVHD
20	Caniche	9	1	T13 - L1	IVHD
21	Pekingese	6.4	8.25	T11 - T12	IVHD
22	Bichon	5.3	3.5	T11 - T12	IVHD
23	Teckel	7.2	6	T11 - T12	IVHD
24	Caniche	9	0.75	T11 - T12	IVHD
25	Chihuahua	1.5	1	T11 - T12	IVHD
26	Chihuahua	2.1	3.25	T13 - L1	IVHD
27	Spitz	6.5	8.5	T13 – L1	IVHD
28	Crossbreed	9.5	7.5	T13 – L1	IVHD
29	Crossbreed	9.7	6	T13 – L1	IVHD
30	Bichon	5.2	3.5	T13 – L1	IVHD
31	Pekingese	7.1	4.75	T13 – L1	IVHD
32	Teckel	7.5	3.25	T13 – L1	IVHD
33	Teckel	5.5	4	T13 – L1	IVHD
34	Crossbreed	7.8	8.5	T13 – L1	IVHD
35	Pomeranian	4	7.25	T13 - L1	IVHD
**Dogs that did not develop spinal walking**					
1	Crossbreed	18.4	10	T10 - T11	Trauma
2	Crossbreed	15.6	8.25	T10 - T11	Trauma
3	Crossbreed	10.2	6.5	T11 - T12	Trauma
4	Bichon	5.5	10.5	T13 - L1	Trauma
5	Teckel	7.3	6	T10 - T11	IVHD
6	Crossbreed	11	2.25	T10 - T11	IVHD

7	Crossbreed	15.7	3	T11 - T12	IVHD
8	Teckel	11.2	3.5	T13 - L1	IVHD
9	Crossbreed	20.4	4.5	T13 - L1	IVHD
10	Crossbreed	40.8	4	T13 - L1	IVHD
11	Crossbreed	45.2	6.75	T13 - L1	IVHD
12	Labrador	41.5	2.25	T11 - T12	IVHD
13	Crossbreed	22.6	3	T11 - T12	IVHD
14	Teckel	10.5	5.5	T11 - T12	IVHD
15	Bichon	9.5	10.75	T11 - T12	IVHD
16	Crossbreed	12.9	4.5	T11 - T12	IVHD
17	Crossbreed	11.5	7	T11 - T12	IVHD
18	Crossbreed	16	5.25	T11 - T12	IVHD
19	Crossbreed	11.7	6	T11 - T12	IVHD
20	Teckel	6.2	6.5	T11 - T12	IVHD
21	Pug	7.4	6	T11 - T12	IVHD
22	Caniche	10.5	3.75	T11 - T12	IVHD
23	Crossbreed	18.7	8.25	T11 - T12	IVHD
24	Crossbreed	20.6	3	T11 - T12	IVHD
25	Crossbreed	18	2.5	T13 - L1	IVHD
