# Supplementary material for: Recovery of Spinal Walking in Paraplegic Dogs Using Physiotherapy and Supportive Devices to Maintain the Standing Position
Source: Animals (Basel). 2023 Apr 19;13(8):1398. doi: 10.3390/ani13081398 (PMC10135265; doi:10.3390/ani13081398)
Supplement: Supplementary file 1 [file animals-13-01398-s001.zip › Table S2.pdf]

Table S2 Data of the general population of dogs involved in the study

Dogs	60
Breeds most represented	Mixed breed (n=25; 31.25%)
	Teckel (n=8; 10%)
	Bichon (n=7; 8.75%)
	Pekingese (n=4; 5%)
	Caniche (n=3; 3.75%)
Age	m: 59.9months (range: 3-129)
Weight	8.27kg (range: 1.5-45.2)
Dogs with IVDE	53 (66.25%)
Dogs with traumatic injuries	7 (8.75%)
Dogs with lesion T9 - T10	29 (36.25%)
Dogs with lesion T10 - T11	6 (7.5%)
Dogs with lesion T11 - T12	22 (27.5%)
Dogs with lesion T13 - L1	29 (36.25%)
Dogs with lesion L1 – L2	1 (1.25%)

m=median; IVDE intervertebral disc extrusion
