# Supplementary material for: Recovery of Spinal Walking in Paraplegic Dogs Using Physiotherapy and Supportive Devices to Maintain the Standing Position
Source: Animals (Basel). 2023 Apr 19;13(8):1398. doi: 10.3390/ani13081398 (PMC10135265; doi:10.3390/ani13081398)
Supplement: Supplementary file 1 [file animals-13-01398-s001.zip › Text S1. Additional explanations about electrostimulation therapy.pdf]

In order to understand the benefits of electrostimulation in veterinary medicine, some basic explanations and definitions of electric current parameters will be briefly presented. There are three types of current commonly used:

1) Direct continuous current (DCC)

2) Direct alternating current (DAC)

3) Pulsating current (PC)

DCC is a unidirectional electric current flowing for one second or more; alternating current changes direction at least once every second. DCC (also called galvanic current) has been used in wound healing, iontophoresis and in the treatment of denervated muscles. In current practice it is only used for iontophoresis. DAC current is not used for therapeutic purposes (Millis et al., 2004, cited by 23). PC (waveform current) is the type of visual representation of current on the x- and y-axis, representing current/time of action or voltage/time of action. They may be symmetrical, asymmetrical, regular or irregular, single-phase, two-phase or multi-phase, and so on.

Amplitude: the value of the current within a single-phase pulse or within a single phase of a two-phase pulse. Cycle/pulse duration: the duration of the cycle or pulse is usually measured in microseconds. Pulse rate is the rate of oscillations in cycles per second, expressed in pulses per second (pps) or hertz (Hz). It is usually categorized as pulse rate or pulses/second, or frequency per stimulus. On/off time is the period of time the machine is transmitting current compared to the rest of the period between contractions and is usually measured in seconds. Ramp is the difference between active stimulation and the pause between stimulation periods. Polarity: the electrode can be anode (+) or cathode (-). Electrical pulsation is represented by the flow of unidirectional or bidirectional electric current, which stops periodically for a defined period of time. Frequency: generally between 25 and 50 Hz (these have been used in human medicine to produce strong tetanic contractions while decreasing the degree of exertion). Waveforms: there are many categories and each of these that is capable of depolarizing muscle fibers is accepted.

Pulse duration and phase duration: between 100 and 400 microseconds. Ramp (rise and fall time): adjust 2-4 seconds longer to increase comfort. On/off time: a ratio of 1:4 or 1:5; 10 seconds on, 40 or 50 seconds off are commonly used. This ratio may decrease with improved muscle strength. A ratio of 1:1, 1:2 or 1:3 is commonly used for increasing muscle strength. Both alternating and direct current are commonly used in neuromuscular electrostimulation devices (Millis & Levine 2013 cited by 23).

Stimulation parameters. Current amplitude (also referred to as magnitude or intensity) is defined as the vertical distance between the highest and lowest point during an electrical stimulus and is commonly measured in milliamperes (mA). Increasing amplitude induces stronger muscle contraction by drawing the electrical current deeper into the muscle, further away from the electrodes (David et al., 2007 cited by 23).

The skin opposes the electrical flow through ohmic resistance and capacitive impedance. Capacitive impedance is dependent on the individual and cannot be altered. Trimming hair from the surface of the skin and cleansing it with alcohol to remove fat or other substances helps to lower the ohmic resistance of the skin. This helps to decrease the voltage required for penetration of the skin by electrical pulses. Increasing the amplitude of the electrical current is necessary to produce some muscle force if the pulse

or phase duration is short. Symmetric and asymmetric biphasic pulsating current use moderate amplitudes. Portable neuromuscular electrostimulation devices are not always capable of producing a current flow similar to fixed ones; however, most commercially available devices used for medical purposes are usually adequate. Pulse durations of 200 to 400 microseconds produce powerful contractions while minimizing the likelihood of muscle fibers pain. Some machines designed solely for neuromuscular electrostimulation do not allow control of pulse duration but will usually fall within this limit. The pulse rate (commonly called pulses per second, pps) is the number of pulses per second and is measured in hertz (Hz). Tetanic muscle contractions can be produced by low frequencies, e.g. 20 Hz, but only the submaxillary muscles can be stimulated at this intensity. The maximum force of contraction generally occurs at 60 to 100 Hz. However, as the frequency increases, so does the rate of muscle exhaustion. Lower frequencies, between 35 and 50 Hz, reduce muscle fatigue while giving strong muscle contractions.

Ramp is a feature of the electrostimulation apart that helps improve patient comfort. It involves gradually increasing or decreasing the amplitude of the current so that the number of motor units gradually increases the strength of the muscle contractions, or gradually decreases the strength of the contractions. The ramp is the period of time during which the electrical pulses increase or decrease in intensity. There are no exact data specifying the optimal ramp duration.

The main criteria in choosing **electrodes** are: flexibility, size, resistance (usually 100 ohms) and increased conductivity. Some commercially available electrodes can be used only a few times, while others can be used up to 100 sessions or more (electrodes coated with carbon-impregnated silicone rubber). The conductivity of any type of electrode decreases with time. Electrodes require a special medium to transmit the electric current. Usually these media are gels, sponges or paper towels; some electrodes already have a medium applied. Sponges and wipes tend to dry out over time, so rewetting them is necessary every 30 minutes. The electrodes must be the right size to stimulate the exact muscle region that therapist want, without the risk of stimulating neighbouring regions. The smaller the electrodes, the greater the density of the current entering the muscles and the greater the pain felt by the animal.
(Millis & Levine 2013, cited by 23)
